# Methodological discrepancies in cancer burden estimates for India

**DOI:** 10.1016/j.lansea.2025.100566

**Published:** 2025-03-26

**Authors:** Sabyasachi Shukla

**Affiliations:** School of Public Health, Kalinga Institute of Industrial Technology (KIIT) Deemed to be University, Bhubaneswar, India, 751024

The study by Singh et al.,^1^ provides an important assessment of India's cancer burden using GLOBOCAN 2022 data. However, several methodological inconsistencies require clarification to ensure data accuracy and reliability. First, the study estimates male and female populations based on a sex ratio of 106.453 males per 100 females, referencing the National Family Health Survey (NFHS-5). Notably, NFHS-5 reports a sex ratio of 98.039 males per 100 females (1020 females per 1000 males),^2^ which may impact crude rate calculations.

Second, the study reports 1,413,316 total cancer cases in 2022. However, applying the reported crude incidence rates (104.5 per 100,000 for females, 91.5 per 100,000 for males) to the respective populations (0.676 billion females, 0.731 billion males) yields an estimate of 1,375,285 cases-a shortfall of 38,031 cases (2.7%). A similar discrepancy exists in mortality estimates, where the calculated total (888,674 deaths) is 28,153 deaths (3.1%) lower than the reported 916,827 deaths.

Third, the study states that cancer incidence in India increased by 36% from 2012 to 2022, reaching 1.38 million cases, with mortality rising by 30.3% to 0.89 million deaths. In contrast, GLOBOCAN 2022 reports 1,413,316 cases and 916,827 deaths,^3^ indicating a further discrepancy.

Finally, while GLOBOCAN 2022 reports 19.97 million global cancer cases and 9.74 million deaths,^4^ data in Supplementary material S2 suggest 18.47 million cases and 8.62 million deaths, a 1.5 million case and 1.12 million death difference requiring clarification. Addressing these inconsistencies will enhance the study's credibility and its contribution to India's cancer surveillance efforts.

## Contributors

SS is the sole author on this manuscript.

## Declaration of interests

None.
